# Uncovering HIV-1-infected cells

**DOI:** 10.18632/oncotarget.4974

**Published:** 2015-07-21

**Authors:** Maxime Veillette, Jonathan Richard, Andrés Finzi

**Affiliations:** Centre de Recherche du CHUM and Department of Microbiology, Infectiology and Immunology, Université de Montréal, Montreal, Quebec, Canada and Department of Microbiology and Immunology, McGill University, Montreal, Quebec, Canada

**Keywords:** Immunology and Microbiology Section, Immune response, Immunity, HIV-1, Env, ADCC, non-neutralizing antibodies, CD4-bound conformation

It has been known for more than 30 years that Human Immunodeficiency Virus 1 (HIV-1) infection drives a very potent B cell response resulting in the production of anti-HIV-1 antibodies targeting several viral proteins, particularly its envelope glycoproteins (Env) which are exposed at the surface of viral particles and infected cells [1]. Enthusiasm by the generation of these anti-Env antibodies was short-lived since the majority were found to be unable to efficiently neutralize viral particles and do not appear to control viral replication. This class of antibodies were therefore called non-neutralizing antibodies. We recently reported that these antibodies, elicited in the majority of HIV-1-infected individuals, do have the potential to eliminate HIV-1-infected cells by an immune mechanism called antibody-dependent cellular cytotoxicity activity (ADCC). However, HIV-1 developed a highly-sophisticated strategy to avoid it [2, 3]. We found that these ADCC-mediating antibodies present in sera [2, 4], breast milk [4] and cervicovaginal lavages [4, 5] of HIV-1-infected individuals preferentially target Env in its CD4-bound conformation. In other words, they only recognize epitopes exposed upon interaction with the viral receptor CD4. In order to avoid ADCC responses, HIV-1 accessory proteins Nef and Vpu decrease the overall amount of Env (via Vpu-mediated BST-2 downregulation) and CD4 at the cell surface [3]. In our view the presence of antibodies with the capacity to eliminate HIV-1-infected cells by Fc-mediated effector functions, including ADCC, represents one of the driving forces for HIV-1-mediated CD4-downregulation. Henceforth, the vast majority of circulating HIV-1 strains worldwide express functional Nef and Vpu proteins, likely limiting the exposure of CD4-induced (CD4i) Env epitopes at the surface of HIV-1-infected cells; thus, protecting them from ADCC-mediated killing.

Can we exploit this information to fight HIV back? As we discussed, the majority of infected individuals do possess antibodies with the potential to eliminate infected cells but the virus “knows” this and avoids exposure of the epitopes being targeted (Env CD4i epitopes). We could hypothesize that strategies aimed at preventing Nef and Vpu-mediated CD4 / BST2 downregulation or designed to “push” Env towards its CD4-bound conformation could potentially increase the susceptibility of HIV-1-infected cells to ADCC and other Fc-mediated effector functions. In a proof-of-concept approach we tested the later possibility by using small-CD4 mimetics and observed that they can indeed force Env to sample the CD4-bound conformation and therefore increase the susceptibility of HIV-1-infected cells to ADCC [4]. In light of these promising results we think that other properties of non-neutralizing antibodies should be highlighted; we believe that non-neutralizing antibodies should be better studied by the HIV-1 scientific community. Unfortunately, their inability to neutralize viral particles did not make them very attractive to HIV-1 researchers so far. Of note, under certain circumstances, such as in the presence of CD4-mimetics, these “non-neutralizing” antibodies can actually neutralize primary viruses [6]. We strongly believe that there is more to an antibody than its neutralization capacity. Through their Fc portion, antibodies can mediate several immunological responses (ADCC, antibody-mediated complement activation, antibody-mediated cellular phagocytosis (ADCP), antibody-dependent cell-mediated virus inhibition (ADCVI), transcytosis inhibition or opsonization) that could be beneficial in fighting viral infections including HIV-1. We think that these important properties should be emphasized and therefore we propose to call them non-neutralizing effector function competent (nNeFC) antibodies.

While we still do not know whether strategies aimed at exposing Env epitopes recognized by nNeFC antibodies will translate into clinical benefits for HIV-1 infected individuals, data generated so far certainly underscores the importance of studying these antibodies in more detail. In the future, nNeFC antibodies through their Fc-effector function might play an important role in the design of new strategies aimed at specifically eliminating HIV-1-infected cells.
